# A Surface Mediated Supramolecular Chiral Phenomenon for Recognition of l- and d-Cysteine

**DOI:** 10.3390/nano8121027

**Published:** 2018-12-10

**Authors:** Jing Wang, Shuai-Shuai Zhang, Xu Xu, Kai-Xuan Fei, Yin-Xian Peng

**Affiliations:** School of Environmental and Chemical Engineering, Jiangsu University of Science and Technology, Zhenjiang 212003, China; zhang7793622@gmail.com (S.-S.Z.); xuxumada@gmail.com (X.X.); fei1567552@gmail.com (K.-X.F.)

**Keywords:** supramolecular chirality, cysteine, silver nanoparticles, mesoporous silica

## Abstract

Chiral recognition is of fundamental importance in chemistry and life sciences and the principle of chiral recognition is instructive in chiral separation and enantioselective catalysis. Non-chiral Ag nanoparticles (NPs) conjugated with chiral cysteine (Cys) molecules demonstrate strong circular dichroism (CD) responses in the UV range. The optical activities of the l-/d-Cys capped Ag NPs are associated with the formation of order arrangements of chiral molecules on the surface of Ag NPs, which are promoted by the electrostatic attraction and hydrogen bonding interaction. The intensity of the chiroptical response is related to the total surface area of Ag NPs in the colloidal solution. The anisotropy factor on the order of 10^−2^ is acquired for Ag NPs with the size varying from ~2.4 to ~4.5 nm. We demonstrate a simple and effective method for the fabrication of a quantitative chiral sensing platform, in which mesoporous silica coated Ag nanoparticles (Ag@mSiO_2_) were used as chiral probes for recognition and quantification of Cys enantiomers.

## 1. Introduction

l-Cysteine (l-Cys), a natural proteinogenic amino acid containing a thiol moiety, plays a key role in biological systems involving protein synthesis, detoxication, and metabolic processes. An abnormal level of l-Cys in the human body is a risk factor for various human diseases. A deficiency of l-Cys is associated with liver damage and skin lesions, and elevated l-Cys concentration is a biomarker for increased risk of cancer and Alzheimer’s disease [1,2]. d-Cys is an essential chiral intermediate for pharmaceuticals. For example, it is a crucial intermediate for the synthesis of cefminox sodium, a broad-spectrum, third-generation cephalosporin antibiotic. d-Cys has also been shown to be a powerful inhibitor of some bacteria, such as *Escherichia coli* [3]. Therefore, developing a powerful new method for accurate identification and quantification of Cys is critically important in the fields of biological systems, pharmaceutical sciences, and biotechnology.

Various methods have been used for the chiral recognition of enantiomers, in particular, optical methods, such as colorimetry [4,5,6,7], spectrofluorimetry [8,9,10,11] and circular dichroism (CD) spectroscopy [12,13,14,15,16,17,18] have received great interest due to their apparent advantages over other methods by virtue of their high sensitivity and the convenience in operation. CD spectroscopy is generally utilized to analyze the molecular chirality, which is sensitive to the conformational changes of the chiral molecules. The CD signal of cysteine appears at the troublesome wavelength of around 200 nm and is intrinsically very weak due to the weak light-molecule interaction. For the conventional CD spectral measurement, a high concentration of Cys is required, restricting the practical application in many aspects. Recently, theoretical and experimental studies revealed that the chiral properties of chiral molecules located at the gaps between the adjacent noble metal nanoparticles (NPs) can be both strongly enhanced and transferred to the visible wavelength due to Coulomb interactions between the chiral molecules and plasmonic NPs [19,20,21,22,23,24]. It has been reported that the chiral recognition of enantiomeric Cys was achieved for the assemblies of Au NPs by monitoring the plasmon enhanced CD response in the Vis range that is related to the chiral nature of the molecule [25,26]. However, the assembly of NP is a complex dynamic process and the disordered NP assemblies lead to a rather weak, even nondetectable, CD signal at the surface plasmon resonance (SPR) frequency. It is difficult for these assemblies of NPs to obtain an accurate and stable chiroptical response in practice due to the uncontrolled nature of the self-assembly process.

Here we report a simple and powerful strategy for the chiral recognition of Cys enantiomers based on the interaction between Ag NPs and the enantiomers. The CD signals in the range of 240–350 nm that are very sensitive to the enantiomeric Cys are used for the chiral discrimination. The origin of the CD signals is related to the surface-mediated ordered assembly of Cys molecules through electrostatic attraction and hydrogen bonding interactions. To explore the application of this CD phenomenon as biosensor, a novel platform is designed using mesoporous silica coated Ag nanoparticles (Ag@mSiO_2_) as a chiral nanosensor. Compared with the unprotected silver NPs, the Ag NP in Ag@mSiO_2_ is protected by a layer of mesoporous silica that can significantly prevent the generation of the unstable CD signals caused by the aggregation of the NPs, thus effectively improving the stability and reliability of the platform, which is of great importance for the practical applications.

## 2. Materials and Methods

### 2.1. Materials

Silver nitrate (AgNO_3_), l-Cysteine (l-Cys), d-Cysteine (d-Cys), sodium citrate (SC), sodium borohydride (NaBH_4_), cetyltrimethylammonium bromide (CTAB) and tetraethoxysilane (TEOS) were obtained from Sigma-Aldrich and used without further purification. Anhydrous ethanol, concentrated HCl (37 wt %) and concentrated ammonia aqueous solution (25 wt %) were purchased from Nanjing Chemical Reagent Co., Ltd (Nanjing, China). Ultrapure water (Millipore, Boston, MA, USA) with a resistivity of 18.2 MΩ·cm was used for all the experiments.

### 2.2. In Situ Synthesis of l-/d-/dl-Cys Capped Ag NPs

l-Cys capped Ag NPs were synthesized by NaBH_4_ reduction of AgNO_3_ in the presence of l-Cys and sodium citrate. In a typical synthesis, 30 μL of AgNO_3_ solution (50 mM), 30 μL of l-Cys solution (50 mM) and 30 μL of sodium citrate solution (0.3 M) were added to 2.9 mL of water and reacted for 30 min under magnetic stirring at room temperature. Then 30 μL of freshly prepared NaBH_4_ solution (0.1 M) was added dropwise into the above mixed solution under vigorous stirring. The samples with other molar ratios of Cys to AgNO_3_ were prepared in the same way by varying the concentration of Cys from 1.5 to 3.0 mM at fixed AgNO_3_ concentration (0.5 mM). The synthesis of d-/dl-Cys capped Ag NPs was similar to the above procedure except that l-Cys was replaced by d-/dl-Cys.

### 2.3. Synthesis of SC-Stabilized Ag NPs

SC-stabilized Ag NPs were synthesized by borohydride reduction of silver nitrate according to a previously described procedure with slight modification [27]. Briefly, 600 μL of AgNO_3_ solution (0.1 M) and 300 μL of SC solution (0.6 M) were first mixed in 55 mL of H_2_O at room temperature. Then 1.5 mL of freshly prepared NaBH_4_ solution (0.1 M) was added dropwise into the mixture containing AgNO_3_ and SC under vigorous stirring.

### 2.4. Synthesis of l-Cys Capped Ag NPs by Ligand Exchange Reaction

l-Cys capped Ag NPs were obtained by ligand exchange of sodium citrate of SC-stabilized Ag NPs with l-Cys at room temperature. In a typical synthesis, 0.9 mL of H_2_O was added to 1 mL of SC-stabilized Ag NPs, followed by the addition of 100 μL of l-Cys solution (10 mM) under magnetic stirring. l-Cys capped Ag NPs were obtained after aging for 12 h at room temperature. The samples with other concentrations of l-Cys were prepared in the same way by varying the concentrations of cysteine from 0.01 μM to 2.5 mM.

### 2.5. Synthesis of Mesoporous Silica Coated Ag Particle (Ag@mSiO_2_)

50 mL of SC-stabilized Ag NPs was concentrated 10-fold by centrifugation and then was added dropwise to 30 mL of CTAB solution (90 mM) under magnetic stirring. After stirring at room temperature for 2 h, the precipitate was collected by centrifugation at 8000 rpm for 30 min, washed with water and redispersed in 1.5 mL of H_2_O. Mesoporous silica coated Ag particles (Ag@mSiO_2_) were synthesized via a surfactant-assembly sol-gel process at room temperature [28,29]. Typically, 13 mg of CTAB was dissolved in the mixed solution containing 3 mL of H_2_O and 2.1 mL of ethanol. Then 1.5 mL of Ag NPs was added dropwise to the above solution under magnetic stirring. After stirring for 5 min, 22 μL of concentrated ammonia aqueous solution (25 wt %) was added. Finally, 92 μL of TEOS was rapidly added under vigorous stirring. After stirring at room temperature for 24 h, the white product was collected by centrifugation at 6000 rpm for 5 min and washed three times with ethanol. To remove the CTAB template, the product was submitted to solvent extraction treatment by dispersing in an ethanol solution (50 mL) containing concentrated HCl (60 μL, 37%), and it was then stirred at 60 °C for 3 h. The sample was collected by centrifugation, followed by washing twice with ethanol, and stored as a powder.

### 2.6. Modification of Ag@mSiO_2_ with Cys

24 mg of Ag@mSiO_2_ was dispersed in 45 mL H_2_O. The obtained Ag@mSiO_2_ suspension was subsequently modified with l-Cys. To 1.75 mL of Ag@mSiO_2_, 250 μL of l-Cys at different concentrations (the final concentration varying in the range of 10~100 μM) was added. The mixtures were aged at room temperature for different times (12, 15, 18, 24 and 48 h). The modification of Ag@mSiO_2_ with d-Cys was similar to the above procedure except that l-Cys was replaced by d-Cys.

### 2.7. Characterization

UV-vis absorption spectra were recorded on a U-3010 spectrophotometer at room temperature. A JASCO J-1500 CD spectropolarimeter with a bandwidth of 1 nm was used to measure the circular dichroism (CD) spectra. A 1 mm quartz cell cuvette was used to measure the absorption and CD spectra of l-Cys, d-Cys and dl-Cys capped Ag NPs, while a 10 mm quartz cell cuvette was used to measure the absorption and CD spectra of l-Cys capped Ag NPs prepared by ligand exchange reaction and Ag@mSiO_2_-l-/d-Cys. Transmission electron microscopy (TEM) images were collected by a JEM-2010 electron microscope operated at an accelerating voltage of 120 kV. The samples for TEM imaging were prepared by dropping the solutions onto carbon-coated copper grids followed by solvent evaporation in air at room temperature.

## 3. Results and Discussions

**Figure 1:**
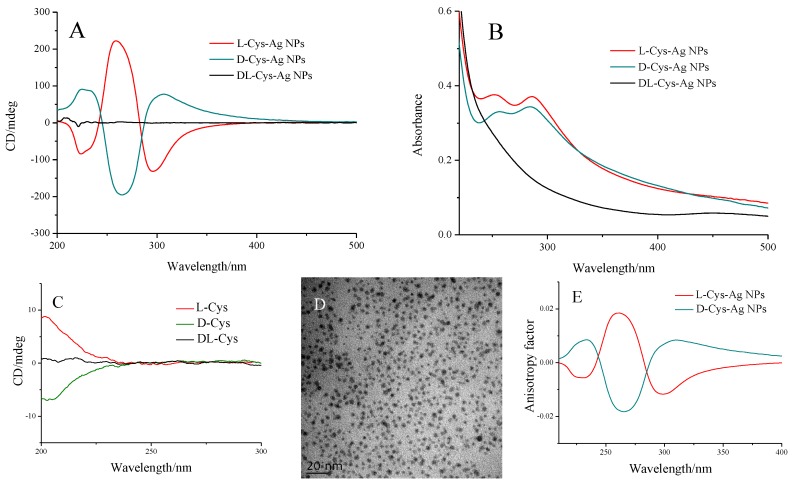
(**A**) Circular dichroism (CD) and (**B**) UV-Vis absorption spectra of l-Cys, d-Cys and dl-Cys capped Ag nanoparticles (NPs); (**C**) CD spectra of l-Cys, d-Cys and dl-Cys solutions; (**D**) Representative transmission electron microscopy (TEM) image of l-Cys capped Ag NPs; (**E**) Anisotropy factors (g-factors) for l-Cys and d-Cys capped Ag NPs.

Cys capped Ag NPs were synthesized by sodium borohydride (NaBH_4_) reduction of the aqueous solution of AgNO_3_ in the presence of cysteine and sodium citrate (SC). l-Cys, d-Cys and dl-Cys capped Ag NPs were obtained by using enantiomeric l-Cys, d-Cys and their mixture in a 1:1 molar ratio as capping agents. Circular dichroism (CD) spectroscopy was utilized to characterize the chiroptical properties of the synthesized Cys capped Ag NPs. As shown in Figure 1A, strong CD signals were observed in the UV range for l-Cys and d-Cys capped Ag NPs, while for dl-Cys capped Ag NPs no CD signal was observed. The CD spectra of l-Cys capped Ag NPs and d-Cys capped Ag NPs showed mirror images with several CD signals in the range of 240–350 nm, which differed from the CD spectra of free l-Cys and d-Cys with mirror images at a wavelength below 250 nm (Figure 1C). The CD response emerging in the UV range was completely different from that of Ag^+^-Cys complexes (Figure S1) [16]. The UV-Vis absorption spectra for l-Cys, d-Cys and dl-Cys capped Ag NPs are shown in Figure 1B. Unlike the Ag nanoparticles with large size, these Cys capped Ag NPs did not exhibit the characteristic surface plasmon resonance (SPR) in the Vis range. The absence of the SPR peak suggested that the synthesized Cys capped Ag NPs had an ultrasmall size. Two absorption peaks at 254 and 285 nm in the UV range were observed for l-Cys and d-Cys capped Ag NPs, which were different from those of Ag^+^-Cys complexes with two absorption peaks at 280 and 357 nm (Figure S1). Figure 1D exhibits the transmission electron microscopy (TEM) image of the synthesized l-Cys capped Ag NPs. It clearly shows that the l-Cys capped Ag NPs had a narrow size distribution with an average diameter of 2.4 nm.

The observed CD response in the UV range was located in the interband electronic transition region of Ag. It has been well demonstrated that optically active thiol protected noble metal nanoclusters display chiroptical properties in the metal-based electronic transition region. In order to compare the chiroptical properties, the anisotropy factor (or g-factor) of Cys capped silver NPs was calculated. Anisotropy factor is defined as g= Δε/ε, where Δε and ε are the molar circular dichroism and molar extinction coefficient, respectively. The g-factor can be estimated by using the following equation: g = θ[mdeg]/(32,980 × A), where θ and A are the ellipticity and the absorbance, respectively. The results are shown in Figure 1E, indicating that the pairs of anisotropy factors of l-Cys and d-Cys capped Ag NPs exhibited a nearly mirror-image relationship in the UV range. The maximum anisotropy factor was ~0.02, which was about one to two orders of magnitude larger than that of the noble metal nanoclusters protected by chiral thiols.

**Figure 2:**
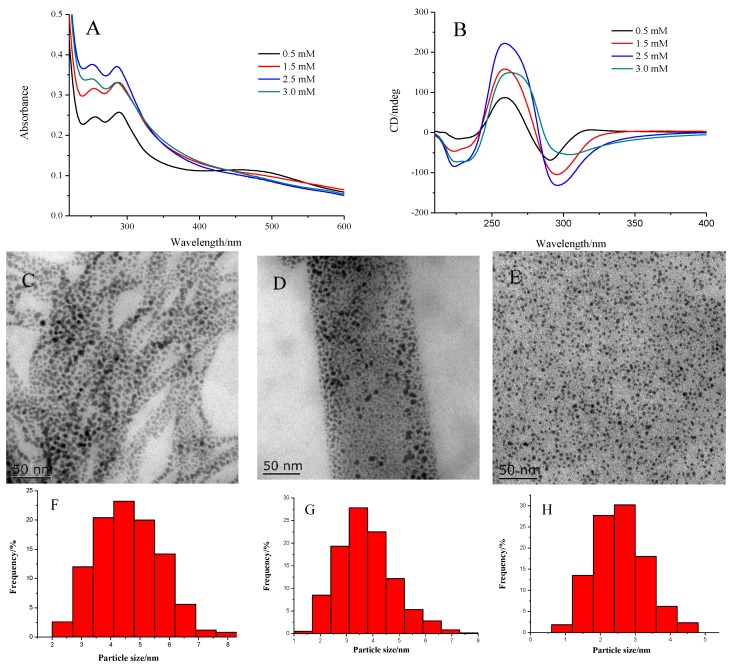
The evolutions of (**A**) UV-Vis absorption and (**B**) circular dichroism (CD) spectra of l-Cys capped Ag nanoparticles (NPs) with the varied concentrations of l-Cys. Transmission electron microscopy (TEM) images of l-Cys capped Ag NPs prepared at different concentrations of l-Cys: (**C**) 0.5 mmol·L^−1^; (**D**) 1.5 mmol·L^−1^ and (**E**) 2.5 mmol·L^−1^; (**F**–**H**) demonstrates the size distribution of l-Cys capped Ag NPs corresponding to (**C**–**E**), respectively.

In order to understand the origin of the chiroptical property of Cys capped Ag NPs, the effect of the Cys concentration on the CD response was explored. Silver NPs were synthesized by varying the concentration of Cys in the mixed solution. The CD and the absorption spectra of Cys capped Ag NPs was measured after aging for ~12 h at room temperature. As shown in Figure 2A, for silver NPs prepared at a low l-Cys concentration (0.5 mM), their absorption spectrum exhibited a typical surface plasmon resonance peak in the Vis range, and two intense peaks in the UV range. The corresponding CD spectrum showed Cotton effects at 260 and 290 nm, respectively (Figure 2B). The obvious mismatch between the spectral region of the observed CD signal and the one expected for the plasmonic chiroptical response ruled out the plasmonic origin of the observed CD signal. With the increase in the concentration of l-Cys from 0.5 to 3.0 mM, the absorptions in the UV range increased, accompanied by a significant decrease in the SPR absorptions in the Vis range. As shown in Figure 2B, the chiroptical responses increased with increasing the concentration of l-Cys, and similar results occurred to the d-Cys capped Ag NPs (Figure S2).

Figure 2C–H shows the typical TEM images together with size distribution histograms of the Ag NPs obtained from the l-Cys concentration of 0.5, 1.5 and 2.5 mM, respectively. In general, nearly spherical nanoparticles can be seen for all the samples, but their sizes were varied. At the l-Cys concentration of 0.5 mM, the average size of Ag NPs obtained was 4.5 ± 1.5 nm. The average size of the Ag NPs decreased from 4.5 to 3.6 and 2.4 nm when the l-Cys concentration increased from 0.5 to 1.5 and 2.5 mM, respectively. The above results revealed that the sizes of the Ag NPs can be tuned by changing the l-Cys concentrations. It was observed that for the synthesis of metal NP, varying the capping agent concentration allowed for size control and with an increase in the concentration of the capping agent, the particle size decreased [30,31]. Cysteine ligand, which has a strong affinity for Ag NPs, can be chemically attached to the surface of Ag NPs through Ag-S bonding. An increased concentration of l-Cys contributed to the effective protection of the formed silver NPs, thus resulting in the formation of Ag NPs with decreased size.

**Figure 3:**
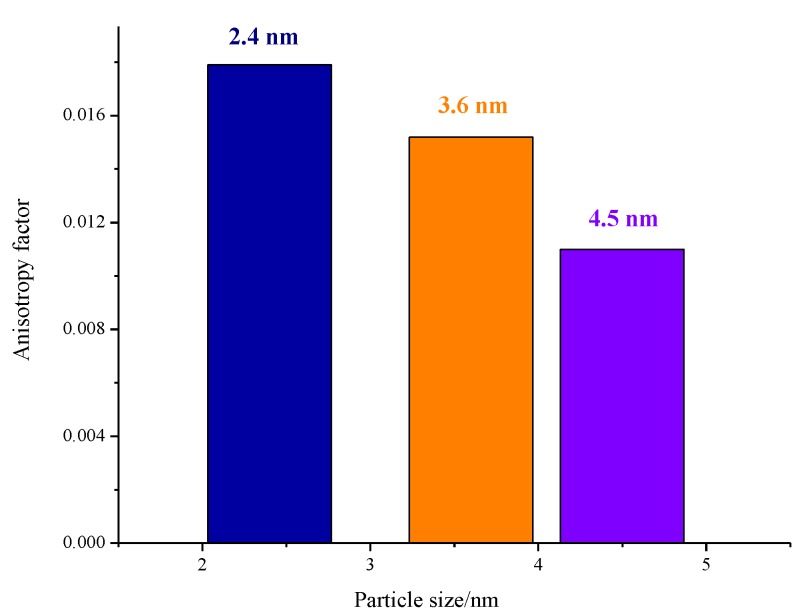
The relationship between the anisotropy factors and the average particle sizes of l-Cys capped Ag nanoparticles (NPs).

For chiral thiol protected noble metal nanoclusters, the chiroptical response in the metal-based electronic transition region was found to strongly decrease with an increase in cluster size [32]. l-Cys capped silver NPs exhibited particle size dependent CD responses. Very intense optical activity in the UV range was observed for Ag NPs with smaller size (Figure 2B). As shown in Figure 3, with an increase in particle size, the anisotropy factor decreased. Yao and co-workers demonstrated the size dependence of anisotropy factors for penicillamine-capped silver and gold nanoclusters [32,33]. The g-factors decreased from 1 × 10^−3^ to 1 × 10^−5^, with an increase in the cluster sizes (from ~1.05 to ~1.86 nm), and the chiroptical property in the metal based transition disappeared at the core diameter of ~2.95 nm. In our case, an anisotropy factor on the order of 10^−2^ was acquired for silver NPs with the size varying from ~2.4 to ~4.5 nm, suggesting a different CD induction mechanism.

**Figure 4:**
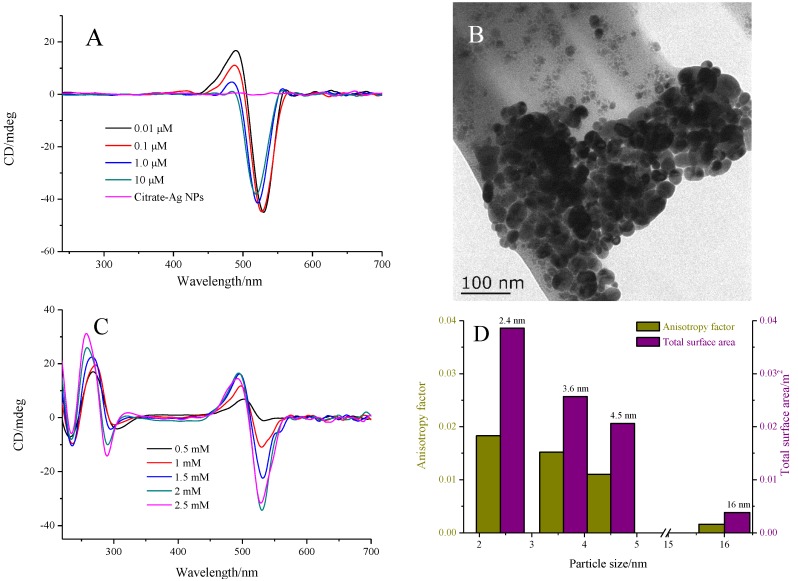
(**A**) The evolutions of circular dichroism (CD) spectra of l-Cys capped Ag nanoparticles (NPs) with the varied concentration of l-Cys; (**B**) The transmission electron microscopy (TEM) image of the aggregated l-Cys capped Ag NPs; (**C**) The evolutions of CD spectra of l-Cys capped Ag NPs with the varied concentration of l-Cys; (**D**) The relationship between the anisotropy factors and the total surface areas of l-Cys capped Ag NPs with the varied particle sizes.

It has been reported that large silver NPs (~20 and ~50 nm in diameter) modified by l-Cys show CD signals in the region from 220 to 320 nm [34]. Two types of silver nanoparticles with different diameters, size distributions and optical parameters exhibited very similar optical activities after incubation with l-Cys. It was concluded that the differences in SPR or in other characteristics of Ag NPs did not affect the CD spectra and Ag NPs just provided suitable substrate for the adsorption of l-Cys. To study the effect of the particle size on the CD signals, the ligand exchange reaction between sodium citrate (SC) and l-Cys was performed on as-prepared SC-stabilized Ag NPs with an average diameter of ~16 nm. l-Cys aqueous solution was added to SC-stabilized Ag NP colloidal solution resulting in a mixture with varied concentration of l-Cys. UV-vis and CD spectra were measured after incubation of the mixtures for 12 h. It was found that with increasing the concentration of l-Cys, the absorption of Ag NPs at 390 nm was slightly decreased, whereas a new peak emerged at 524 nm and its intensity was gradually enhanced (Figure S3). This indicated that the aggregations of Ag NPs occurred upon addition of l-Cys to the colloidal solution. The aggregation of Ag NPs was assisted by the interactions of l-Cys molecules that were adsorbed on the surfaces of Ag NPs through electrostatic attraction and hydrogen bonding. The CD spectra of l-Cys capped Ag NPs prepared by the ligand exchange reaction were shown in Figure 4A. A bisignate CD signal with a positive Cotton effect at 490 nm and a negative Cotton effect at 524 nm was observed. The characteristic bisignate of the CD signal suggests the existence of plasmonic dipole-dipole interactions between adjacent Ag NPs in the aggregates. The appearance of chiroptical responses at the plasmonic spectral region has been observed for metal NPs capped with chiral molecules [27,35,36,37,38,39,40,41,42]. The produced plasmonic CD signals were attributed to the Coulomb interaction between chiral molecules and plasmonic nanoparticles that was greatly amplified by the enhanced electromagnetic fields between adjacent Ag NPs. No CD signals were observed in the UV range because of the lower concentration of l-Cys in the colloidal solution.

When the concentration of l-Cys in the Ag colloidal solution was further increased, the peak at 390 nm dramatically decreased, whereas the peak at 524 nm was significantly enhanced as shown in Figure S4, indicating that the degree of particle aggregation in the colloidal solution was enhanced. Figure 4B shows the TEM image of l-Cys capped Ag NPs prepared by the ligand exchange reaction, showing that the increase in the concentration of l-Cys leads to the obvious aggregation of Ag NPs, which is in accordance with the result of the UV-Vis absorption spectra. The corresponding CD spectrum showed two Cotton effects at 260 and 290 nm in the UV range besides the CD signal at the SPR region (Figure 4C). With the increase of l-Cys concentration, the CD signals gradually increased. The CD signals observed in the region from 240 to 350 nm were identical in shape (not in intensity) with those of l-Cys capped Ag NPs in situ, prepared with ultrasmall sizes (2.4 to 4.5 nm) (Figure 2B). The above results indicate that the particle sizes had no effect on the electronic transitions of l-Cys capped Ag NPs.

Recently, Lei et al. investigated the interaction of Cys with Au@Ag core–shell nanocuboids by CD spectroscopy [43]. A bisignate CD signal was observed in the region of 240–320 nm. It was demonstrated that the observed CD response came from the formation of helical networks of neighboring cysteine molecules that were chemisorbed on the Ag surface through hydrogen bonding between the COO^−^ and NH_3_^+^ groups. A theoretical study showed that Cys molecules are adsorbed on the surface of Au as zwitterionic Cys at high coverage and are stabilized by the formation of intermolecular hydrogen bonds [44]. To investigate the role of hydrogen bonding in inducing the generation of the CD signal, experiments were performed with l-Cys derivatives N-acetyl-l-cysteine and l-cysteine methyl ester. The result of the CD spectra revealed that N-acetyl-l-cysteine and l-cysteine methyl ester-modified Ag NPs did not exhibit chiral responses in the range of 240–350 nm (see Figure S5). For l-cysteine methyl ester (or N-acetyl-l-cysteine) molecules chemisorbed on the Ag surface, intermolecular hydrogen bonding cannot form because the hydrogen atom of the COOH (or NH_2_) is substituted by methyl (or acetyl). The results suggest that the presence of the intermolecular hydrogen bonding among the neighboring chiral ligands that is crucial for the formation of the chiral supramolecular networks on the surface of Ag NPs is necessary for the generation of the CD signal.

Similar CD responses observed for different systems indicated that the CD response in the UV range was irrelevant to the size or the shape of the support material and the origin of the chirality was related to the chiral supramolecular networks of Cys molecules formed on the surface of the support material. Although the particle size of Ag NPs was irrelevant to the shape of the CD signals, the particle size had a significant effect on the anisotropy factor as shown in Figure 4D. With increasing the particle size from 2.4 to 16 nm, the anisotropy factor decreased from the order of 10^−2^ to 10^−3^. In fact, the concentration of Cys in the chiral supramolecular networks on the surface of Ag NPs determined the intensity of the CD signal observed in the UV range. When the concentration of cysteine was enough to form a monolayer on the surface of Ag NPs, the concentration of cysteine adsorbed on the surface was related to the total surface area of the Ag NPs. The total surface area of the Ag NPs (ANPs) with different sizes can be calculated using equation [34]:

$$A_{\mathrm{NPs}}=\frac{3c_{\mathrm{AgNO}3}VM_{\mathrm{Ag}}}{\rho_{\mathrm{Ag}}r_{\mathrm{NP}}}$$

where *c*_AgNO3_ is the concentration of AgNO_3_ solution used for Ag NP preparation, *V* is the volume of the sample, *M*_Ag_ is molecular weight of silver, *ρ*_Ag_ is the density of silver and *r*_NP_ is the radius of a nanoparticle. Figure 4D is a summary of the anisotropy factors and the total surface areas for Ag NPs with different sizes. As can be seen, with an increase in particle size, the total surface area of the Ag NPs gradually decreased. The same trend was observed for anisotropic factor, that is, it decreased with increasing particle size. This suggests that anisotropy factor actually depended on the total surface area of the Ag NPs, as long as the concentration of cysteine is sufficient for the formation of a monolayer on the surface.

**Figure 5:**
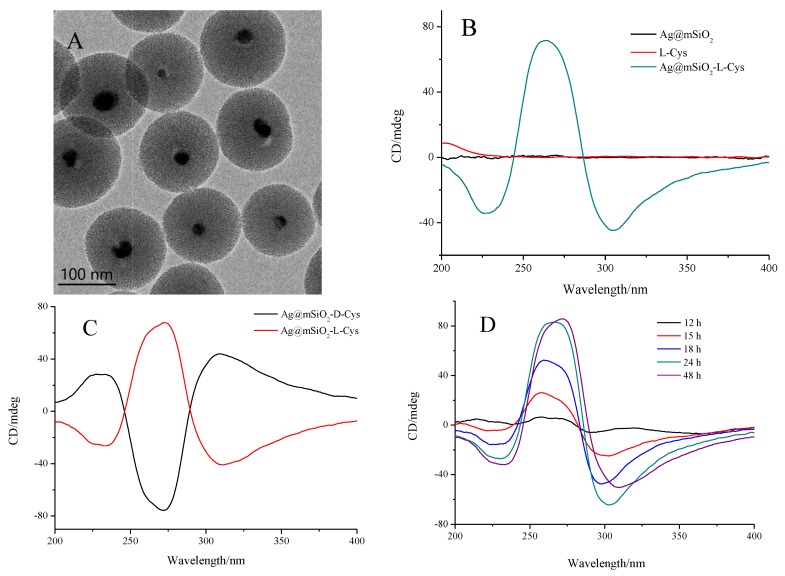
(**A**) Transmission electron microscopy (TEM) image of the mesoporous silica coated Ag nanoparticles (NPs) (Ag@mSiO_2_) after etching; (**B**) The circular dichroism (CD) spectra of Ag@mSiO_2_, l-Cys solution and Ag@mSiO_2_-l-Cys; (**C**) CD spectra of Ag@mSiO_2_-l-Cys and Ag@mSiO_2_-d-Cys; (**D**) The evolutions of CD spectra of Ag@mSiO_2_-l-Cys with the reaction time.

**Scheme 1:**
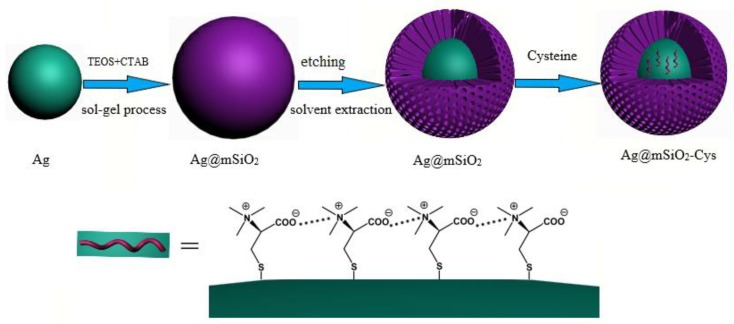
Illustration of the synthesis process for Ag@mSiO_2_ and application for cysteine detection.

The observed CD response in the UV range for Cys capped Ag NPs can be used to recognize and quantify Cys enantiomers. A chiral discrimination platform was designed based on Ag NP as the support material for the adsorption of Cys. To improve the stability of the platform, Ag NPs were replaced by the mesoporous silica coated Ag NPs (Ag@mSiO_2_), as Cys capped Ag NPs were not stable enough in the solution (Figure S6). The overall synthesis procedure of Ag@mSiO_2_ and application for Cys detection are depicted in Scheme 1. The TEM images of Ag@mSiO_2_ before solvent extraction are shown in Figure S7A, demonstrating that the Ag core (~20 nm in diameter) was coated with a layer of mesoporous SiO_2_ shell (~50 nm in thickness). Figure 5A shows the TEM images of Ag@mSiO_2_ after solvent extraction. It can be seen that the Ag core was slightly etched during the process of solvent extraction. It is known that Ag NPs can be oxidized and etched by O_2_ and HCl [45,46]. Solvent extraction was carried out in an ethanol solution containing concentrated HCl at 60 °C. The standard reduction potential of O_2_ is dependent on the pH value of the solution. It can be dramatically enhanced by acid from 0.401 to 1.229 V (versus the normal hydrogen electrode, NHE), while the existence of Cl^−^ can reduce the reduction potential of Ag(I)/Ag(0) from 0.7996 to 0.2223 V (versus NHE) [47]. Therefore, the oxidative etching of Ag NPs can easily take place during the solvent extraction process. The presence of Ag(I) can be further confirmed by its reaction with l-Cys. Upon the addition of l-Cys to Ag@mSiO_2_ after solvent extraction, the interaction of Ag(I) with l-Cys occured immediately, which was indicated by the appearance of the weak CD signal of the Ag^+^-l-Cys complexes (Figure S7B). The etching of the silver cores is advantageous for the adsorption of l-Cys on their surfaces.

After CTAB molecules were removed from Ag@mSiO_2_ and the silver cores were slightly etched, l-Cys aqueous solution was added to Ag@mSiO_2_ suspension and incubated for different times at room temperature. Figure 5B shows the comparison of the CD spectrum of Ag@mSiO_2_ before and after incubating with l-Cys for ~18 h, showing that a bisignate CD signal emerged in the range of 240–350 nm after incubating with l-Cys, which is similar to that of l-Cys capped Ag NPs, whereas no CD response emerged before incubation, which is consistent with the achirality of Ag@mSiO_2_. This indicates that during the incubation, l-Cys molecules diffused through the channels of mesoporous silica and adsorbed on the surface of the Ag core of Ag@mSiO_2_. A perfect mirror image is obtained by using Ag@mSiO_2_-d-Cys as shown in Figure 5C. The interaction of l-Cys with Ag@mSiO_2_ during the process of the incubation was monitored by the UV-vis and CD spectroscopies. As shown in Figure S8A, the SPR peak of the Ag@mSiO_2_ decreased in intensity with the incubation time due to the adsorption of l-Cys on the Ag core, which caused a decrease in the local effective refractive index. Figure 5D shows the evolution of the CD spectrum of Ag@mSiO_2_-l-Cys with the reaction time, revealing that the CD response increased with the reaction time and reached saturation after ~24 h incubation. As shown in Figure S8B, the intensity of the CD signal at 260 nm increased linearly with reaction time, following the first-order adsorption kinetics. The rate constant k was acquired from the slope of the line and it was found to be 3.3 h^−1^.

**Figure 6:**
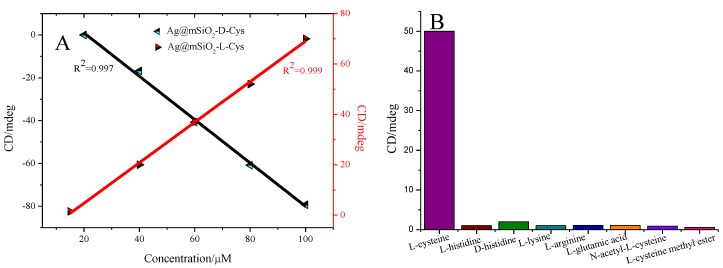
(**A**) The intensity of the circular dichroism (CD) signal at 265 nm as a function of the concentration of l-Cys and d-Cys; (**B**) CD responses of Ag@mSiO_2_ to amino acids at 265 nm. The concentration of each amino acid was 1 mM.

The observed CD signals based on the supramolecular interactions between Cys molecules adsorbed on the Ag core were very sensitive to the Cys enantiomers, making it possible to sense Cys using these CD signals. As shown in Figure S9, with increasing concentrations of l-Cys, the CD signals of Ag@mSiO_2_-l-Cys increased gradually before the adsorption saturation was reached. For Ag@mSiO_2_-d-Cys, the CD signals decreased gradually with increasing concentrations of d-Cys as shown in Figure S10. Figure 6A reveals that good linear relationships within the 20–100 μM concentration range were established between the intensities of CD signals at 265 nm and the concentrations of l-/d-Cys. This demonstrates that the platform established was a highly sensitive chirality sensor, providing a new chiral sensing method to qualify Cys enantiomers. By using the novel chiral discrimination platform, l-Cys molecules as low as ~1.25 × 10^−5^ M can be detected (Figure S11), which cannot be achieved by the conventional electronic CD measurement technique.

To demonstrate the selectivity of the chiral sensing platform, other amino acids were tested in order to evaluate their binding abilities with Ag@mSiO_2_ and the induced chiroptical properties. As shown in Figure 6B and Figure S12, no obvious CD signals at 265 nm were observed as other amino acids were introduced into the Ag@mSiO_2_ suspensions. The sensing platform established can distinguish cysteine enantiomers with high sensitivity and selectivity.

## 4. Conclusions

In conclusion, we report herein an unusual optical activity induced in l-/d-Cys capped Ag NPs, showing a strong mirror image in the UV range. The origin of the chiroptical property is ascribable to the formation of chiral supramolecular networks of chemisorbed l-/d-Cys molecules through electrostatic attraction and hydrogen bonding on the surface of Ag NPs. It was found that the size of Ag NPs did not affect the shape of the CD signals induced. The anisotropy factor increased with an increase in the total surface area of the Ag NPs, when the l-Cys molecules reached the adsorption saturation on their surface. The largest anisotropy (~0.02) was obtained in the system of l-Cys capped Ag NPs with particle size of ~2.4 nm. These chiral recognition phenomena enabled us to establish a chiral sensing platform for chiral discrimination of Cys enantiomers. The platform based on Ag@mSiO_2_ with the function for chiral discrimination is anticipated to be a novel chiral sensor for applications in the fields of asymmetric chemistry, biomedicine, and pharmaceutics.

## Supplementary Materials

The following are available online at http://www.mdpi.com/2079-4991/8/12/1027/s1. Figure S1: (A) CD and (B) UV-Vis absorption spectra of l-Cys, l-Cys+AgNO_3_ and l-Cys capped Ag NPs. Figure S2: (A) CD and (B) UV-Vis absorption spectra of the d-Cys capped Ag NPs prepared at different concentrations of d-Cys. Figure S3: The evolutions of UV-Vis absorption spectra of l-Cys modified Ag NPs with the varied concentration of l-Cys. Figure S4: The evolutions of UV-Vis absorption spectra of l-Cys modified Ag NPs with the varied concentration of l-Cys. Figure S5: CD spectra of Ag NPs in the presence of N-acetyl-l-cysteine and l-cysteine methyl ester. Figure S6: (A) CD and (B) UV-Vis absorption spectra of the l-Cys capped Ag NPs after aging for different time. Figure S7: (A) TEM image of the mesoporous silica coated Ag NP (Ag@mSiO_2_) before etching. (B) CD spectra of the mixture of Ag@mSiO_2_ after etching and l-Cys. Figure S8: The evolutions of (A) UV-Vis absorption spectra of Ag@mSiO_2_-l-Cys with the reaction time. (B) Plot of the CD intensity at 260 nm vs. the reaction time. Figure S9: The evolution of the CD spectra of Ag@mSiO_2_-l-Cys with the varied concentration of l-Cys. Figure S10: The evolution of the CD spectra of Ag@mSiO_2_-d-Cys with the varied concentration of d-Cys. Figure S11: The CD spectra of Ag@mSiO_2_-l-Cys with the concentration of l-Cys of 1.25 × 10^−5^ M. Figure S12: The CD responses of Ag@mSiO_2_ in the presence of amino acids.
