# Supplementary material for: A Surface Mediated Supramolecular Chiral Phenomenon for Recognition of l- and d-Cysteine
Source: Nanomaterials (Basel). 2018 Dec 10;8(12):1027. doi: 10.3390/nano8121027 (PMC6315486; doi:10.3390/nano8121027)
Supplement: Supplementary file 1 [file nanomaterials-08-01027-s001.pdf]

Supporting Information

A Surface Mediated Supramolecular Chiral Phenomenon for Recognition of L- and D-Cysteine

Jing Wang *, Shuai-Shuai Zhang, Xu Xu, Kai-Xuan Fei and Yin-Xian Peng *

School of Environmental and Chemical Engineering, Jiangsu University of Science and Technology, Zhenjiang 212003, China; zhang7793622@gmail.com (S.-S.Z.); xuxumada@gmail.com (X.X.); fei1567552@gmail.com (K.-X.F.)

* Correspondence: wangjingalice@just.edu.cn (J.W.); pyxhx@just.edu.cn (Y.-X.P.); Tel.: +86-(511)-8440-1181 (J.W.); +86-(511)-8563-5850 (Y.-X.P.)

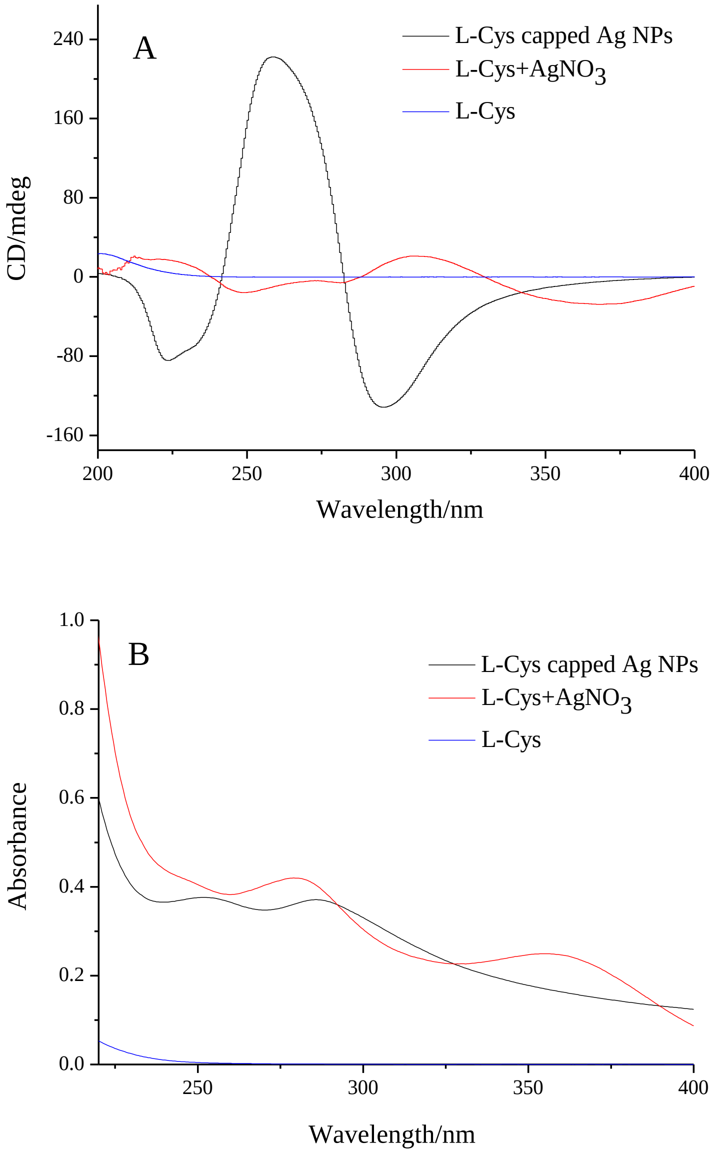

Figure S1 (A) CD and (B) UV-Vis absorption spectra of L-Cys, L-Cys+$AgNO_3$ and L-Cys capped Ag NPs.

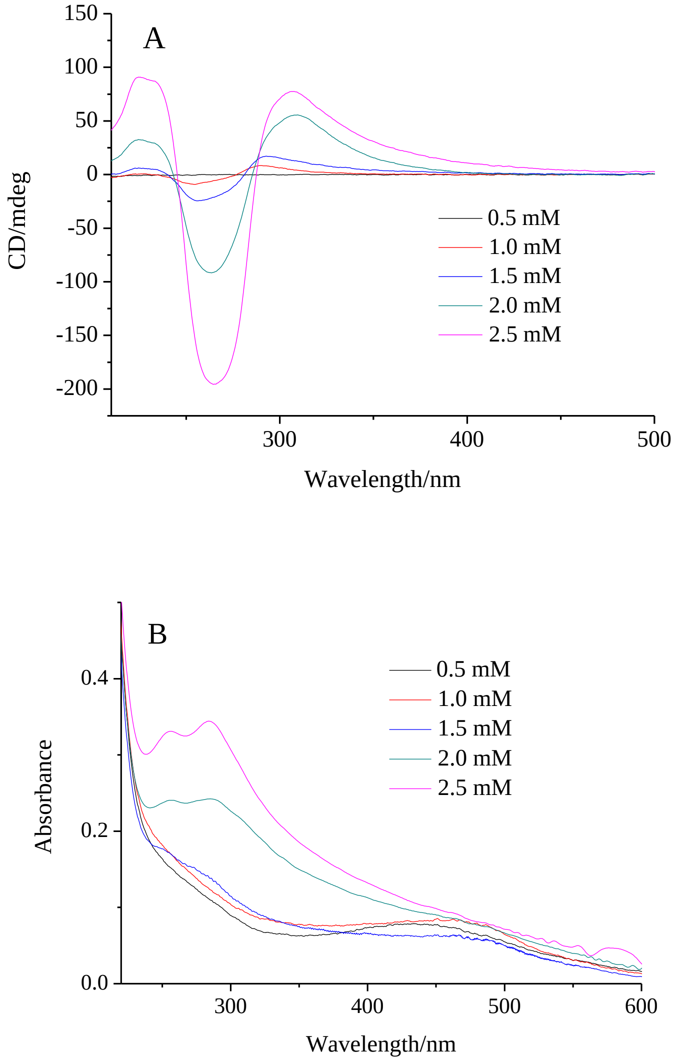

Figure S2 (A) CD and (B) UV-Vis absorption spectra of the D-Cys capped Ag NPs prepared at different concentrations of D-Cys.

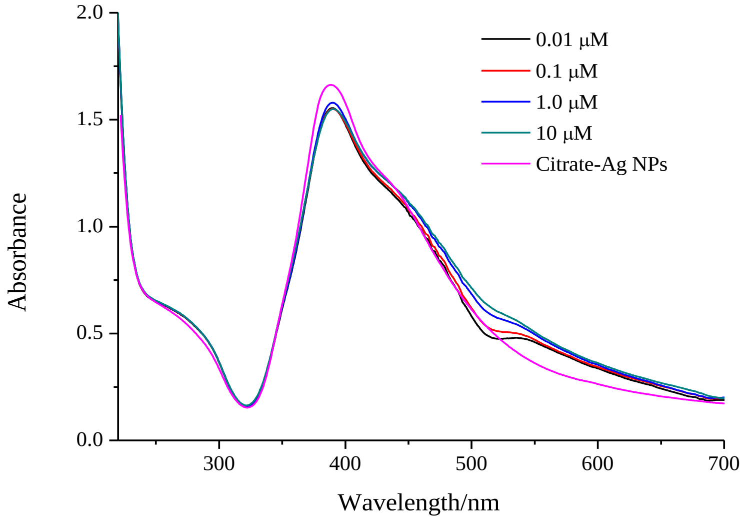

Figure S3 The evolutions of UV-Vis absorption spectra of L-Cys modified Ag NPs with the varied concentration of L-Cys.

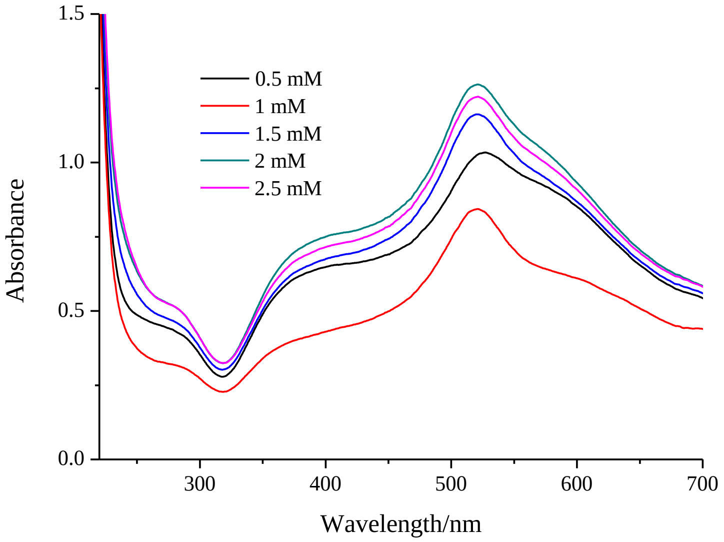

Figure S4 The evolution of the UV-Vis absorption spectra of L-Cys modified Ag NPs with the varied concentration of L-Cys.

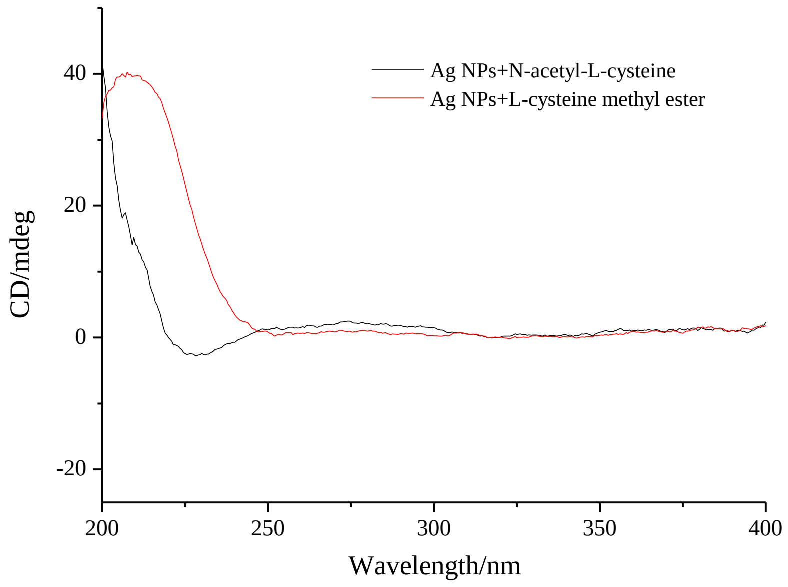

Figure S5 CD spectra of Ag NPs in the presence of N-acetyl-L-cysteine and L-cysteine methyl ester.

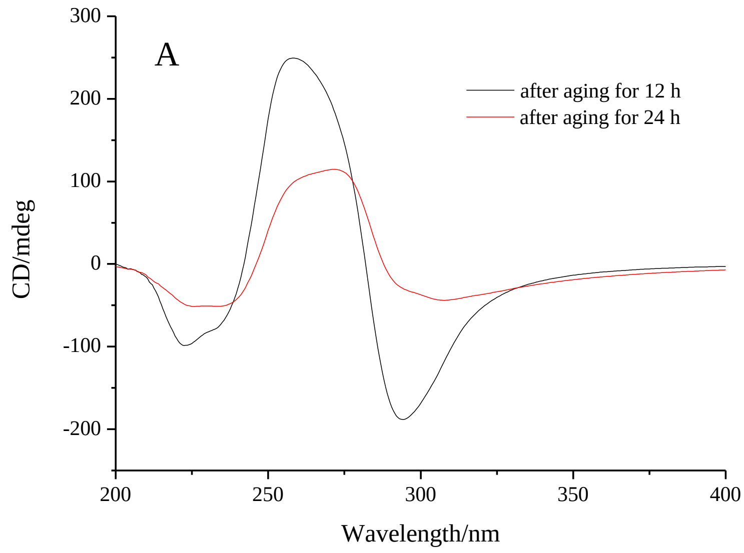

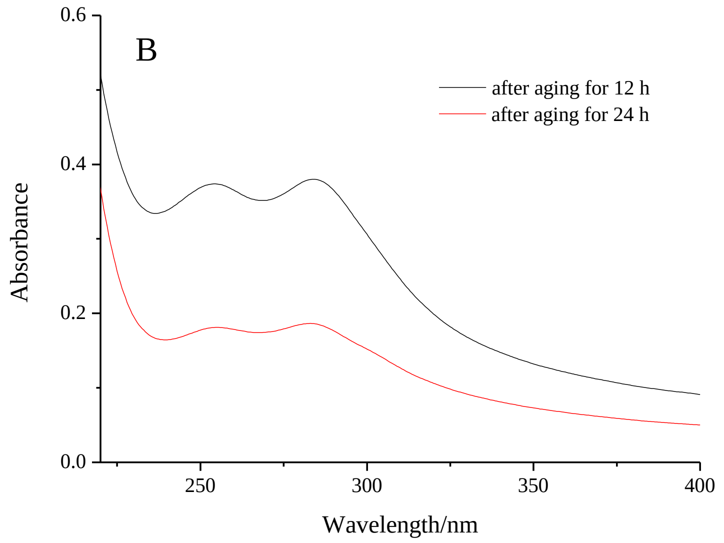

Figure S6 (A) CD and (B) UV-Vis absorption spectra of the L-Cys capped Ag NPs after aging for different time.

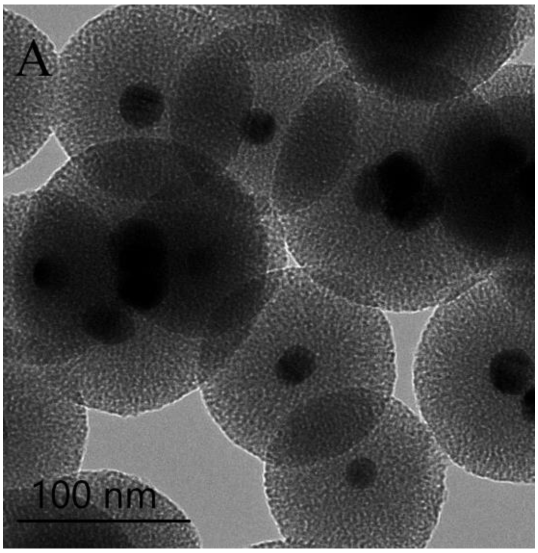

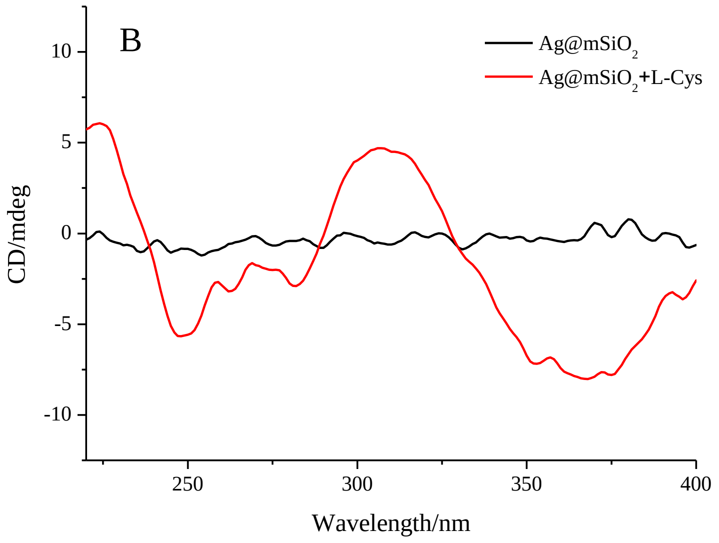

Figure S7 (A) TEM image of the mesoporous silica coated Ag NP ($Ag@mSiO_2$) before etching. (B) CD spectra of the mixture of $Ag@mSiO_2$ after etching and L-Cys.

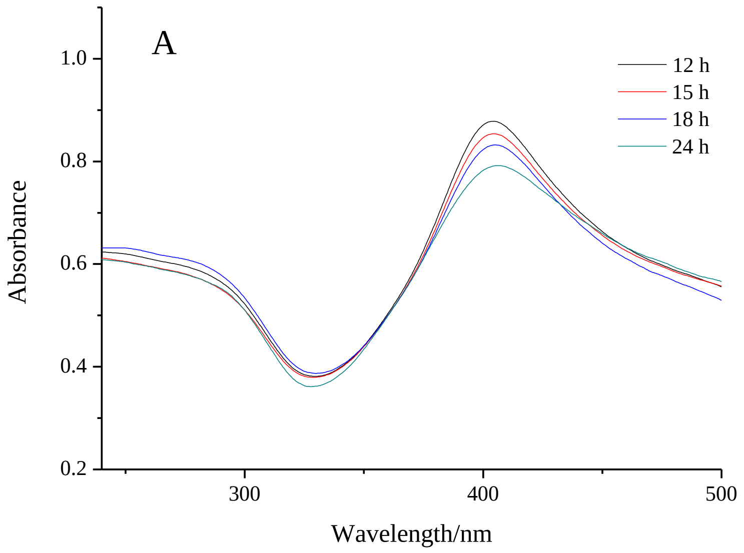

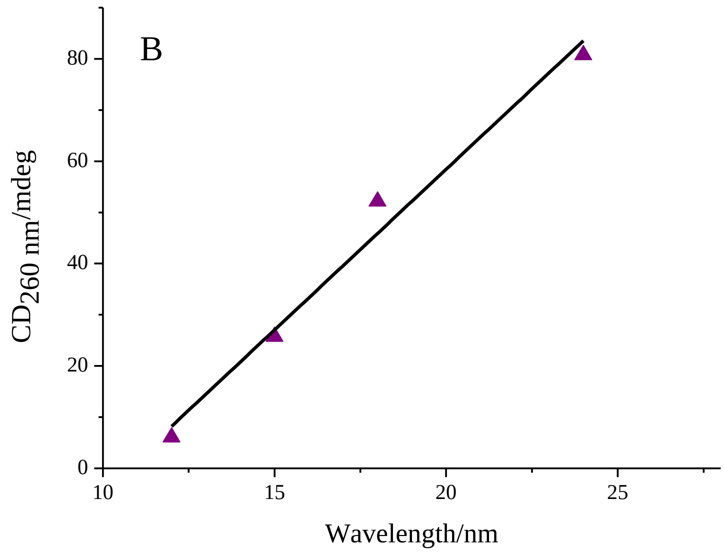

Figure S8 The evolutions of (A) UV-Vis absorption spectra of Ag@mSiO$_2$-L-Cys with the reaction time. (B) Plot of the CD intensity at 260 nm vs. the reaction time.

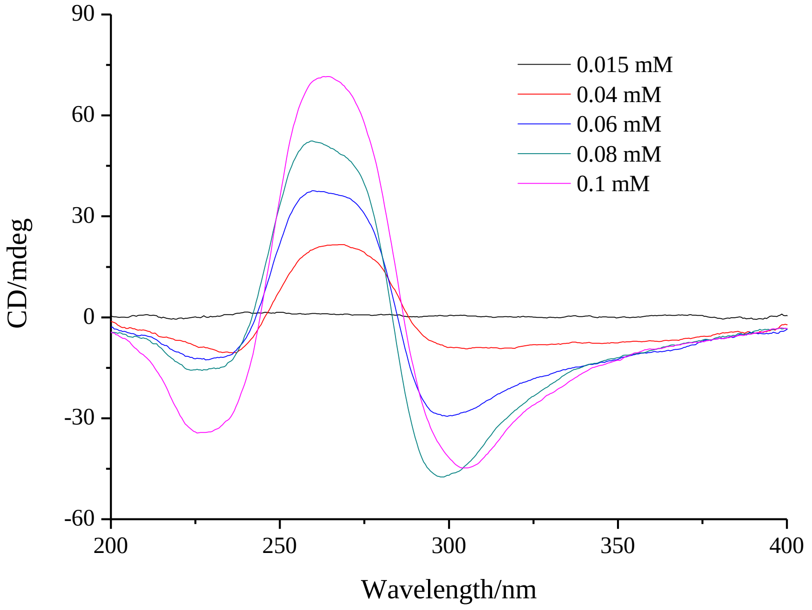

Figure S9 The evolution of the CD spectra of Ag@mSiO$_2$-L-Cys with the varied concentration of L-Cys.

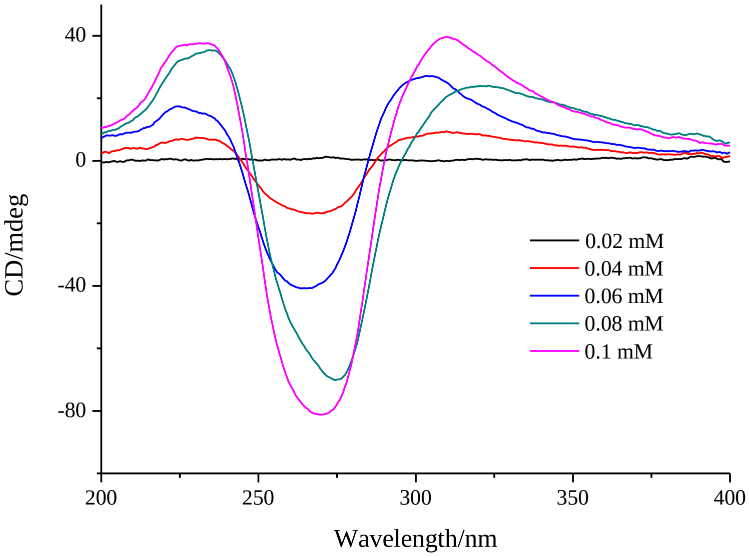

Figure S10 The evolution of the CD spectra of Ag@mSiO$_2$-D-Cys with the varied concentration of D-Cys.

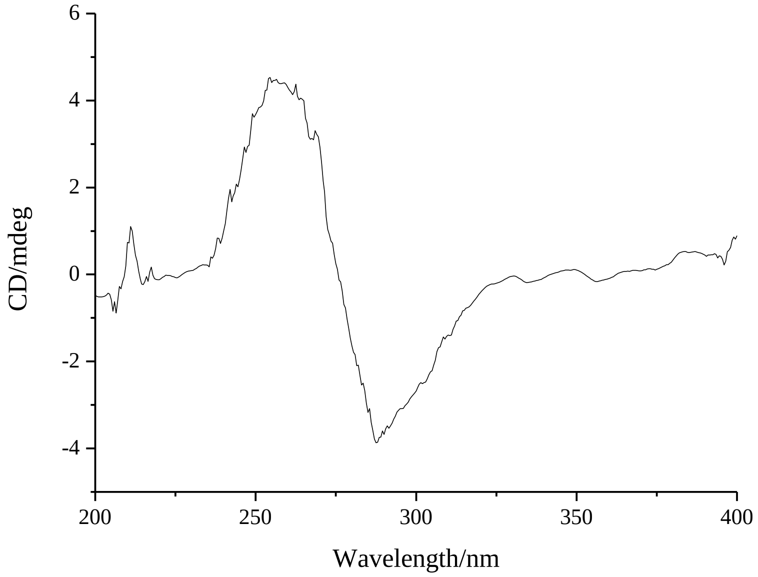

Figure S11 The CD spectra of Ag@mSiO$_2$-L-Cys with the concentration of L-Cys of 1.25×10^{-5} M.

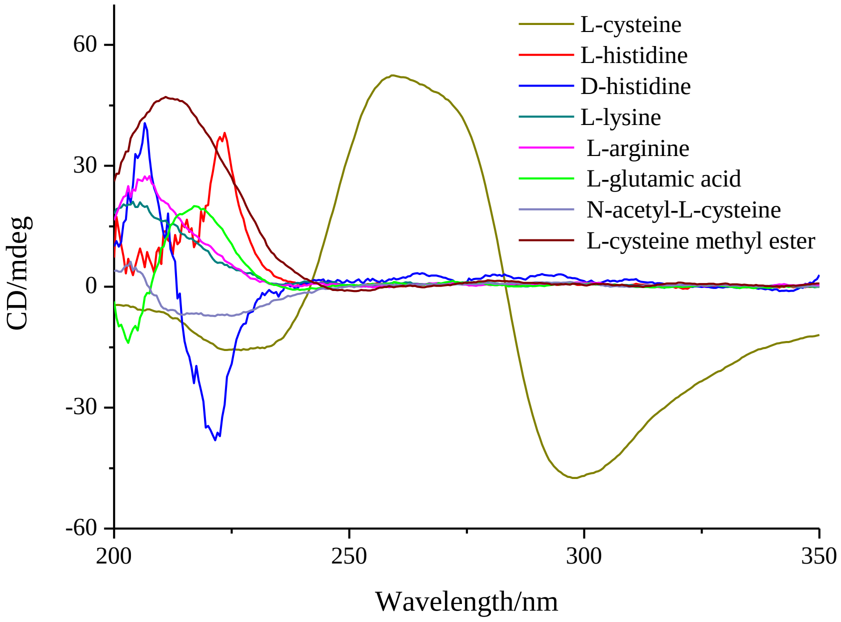

Figure S12 The CD responses of $Ag@mSiO_2$ in the presence of amino acids.
